# Sleep apnea in men is associated with altered lipid metabolism, glucose tolerance, insulin sensitivity, and body fat percentage

**DOI:** 10.1007/s12020-020-02369-3

**Published:** 2020-06-19

**Authors:** Prasad G. Kamble, Jenny Theorell-Haglöw, Urban Wiklund, Karl A. Franklin, Ulf Hammar, Eva Lindberg, Jan W. Eriksson

**Affiliations:** 1 Department of Medical Sciences, Clinical Diabetology and Metabolism, Uppsala University, Uppsala, Sweden; 2 Department of Medical Sciences, Respiratory, Allergy and Sleep Research, Uppsala University, Uppsala, Sweden; 3 Department of Radiation Sciences, Biomedical Engineering, Umeå University, Umeå, Sweden; 4 Department of Surgical and Perioperative Sciences, Surgery, Umeå University, Umeå, Sweden

**Keywords:** Obstructive sleep apnea, Glucose metabolism, Lipid metabolism, Insulin secretion

## Abstract

**Purpose:**

Obstructive sleep apnea (OSA) is associated with obesity and risk for type 2 diabetes. In this community-based study, we thoroughly investigated fatty acid metabolism, incretin response, glucose tolerance, insulin secretion and insulin sensitivity, and autonomic nerve activity in men with or without OSA.

**Methods:**

Fifteen men without diabetes but with signs of severe OSA, defined as apnea–hypopnea index (AHI) >30, and 15 age- and BMI-matched men without OSA (AHI < 5) were recruited from a community-based cohort. Assessments included clinical and anthropometric measurements, a 2-h oral glucose tolerance test (OGTT), and autonomic nerve activity using heart rate variability (HRV).

**Results:**

Men with OSA had higher body fat % than BMI-matched men without OSA (*p* = 0.046) and it was associated with markers of insulin resistance. The area under the curve for nonesterified fatty acids (NEFA) during OGTT was higher in men with OSA (*p* = 0.021) and fasting NEFA levels were numerically higher (*p* = 0.097). The plasma glucose at fasting and during OGTT was higher in men with OSA (*p* < 0.001). Incretin response was similar between groups. Fasting and OGTT-derived indices indicated impaired insulin sensitivity in men with OSA. Compared with men without OSA, Matsuda index (*p* = 0.068) and Gutt index (*p* < 0.01) were lower in men with OSA. The HRV measures did not differ between groups.

**Conclusions:**

Our study suggests that fatty acid handling, glucose tolerance, and insulin sensitivity are impaired in men with severe OSA. This might partly be explained by the increased body fat percentage.

## Introduction

Obstructive sleep apnea (OSA) is characterized by repetitive narrowing causing hypopnea and collapse causing apnea of the upper airway during sleep, leading to oxyhemoglobin desaturation events, recurrent arousal from sleep, and sleep fragmentation [1]. Based on the mean number of apneas and hypopneas per hour of sleep (the apnea–hypopnea index, AHI), OSA is categorized into mild (AHI 5–<15), moderate (AHI 15–<30), or severe (AHI >30) [2]. Even mild OSA is an independent risk factor for cardiovascular outcomes, such as arterial hypertension, ischemic heart disease, arrhythmias, and ischemic stroke [3, 4]. The metabolic abnormalities associated with OSA include defective lipid and glucose metabolism [5], atherogenesis, and abnormal liver function [6, 7]. In addition to intermittent hypoxia, an autonomic imbalance has also been reported to be involved in cardiovascular risk associated with OSA [8].

Data from previous research suggest that OSA, independent of obesity, can cause metabolic disturbances through the effects of sleep fragmentation, intermittent hypoxia, sympathetic overactivity, and adipose tissue inflammation [9, 10], and several epidemiological studies have reported associations between OSA and impaired glucose metabolism [11, 12]. In addition to intermittent hypoxia, an autonomic imbalance has also been reported to be involved in cardiovascular risk associated with OSA. Some data also indicate that OSA during rapid eye movement (REM) sleep is more important than nonrapid eye movement when it comes to diabetes risk [13]. Furthermore, REM-AHI is also associated with elevated sympathetic activity and adverse cardiovascular events in patients with OSA [14, 15].

Moreover, OSA shares considerable overlap with other risk factors for metabolic disturbances including middle age and obesity limiting the possibilities for conclusions on the causal relationship. A deeper understanding of the underlying mechanisms by which OSA influences metabolic function might yield improved therapeutic approaches and outcomes in these patients. The measures of metabolism have previously been compared between patients with OSA vs. individuals without OSA, as well as the effects of OSA treatment in randomized control trials [16]. However, studies thoroughly assessing glucose and lipid metabolism in individuals with OSA identified by community-based screening are lacking. In the present study, we performed a comprehensive investigation of fatty acid metabolism, insulin and incretin response to OGTT, glucose tolerance, insulin sensitivity, and autonomic nervous system activity (heart rate variability, HRV) in men found to have severe OSA compared with men without sleep apnea, and this was done under normal daytime conditions regardless of recent OSA episodes. We also assessed the association between OSA in REM sleep and metabolic measures.

## Material and methods

### Participants

This is a substudy of an ongoing cohort study “Men in Uppsala; a Study of sleep, Apnea and Cardiometabolic Health” (MUSTACHE). In the MUSTACHE study men living in Uppsala and <80 years who had participated in the community-based study Epi-Health are recruited for a comprehensive clinical investigation including questionnaires, polysomnography, anthropometric measurements, and blood samples [17]. For the current study, we performed polysomnography to assess the presence of OSA. The scoring of sleep and associated events were done as per specifications by the American Academy of Sleep Medicine and no questionnaires were used [18]. Based on the obtained scores, the men were then classified into two groups based on their AHI, such as participants without OSA (AHI < 5) or participants with OSA (AHI > 30). All participants with an AHI < 5 were listed with age and BMI. As soon as a participant was identified with an AHI of >30, a control subject (man without OSA) matched for age and BMI was selected. In total 30 participants of which 15 were participants without OSA (mean AHI 3.0 ± SD 1.9) and 15 were participants with OSA (AHI 39.5 ± 7.3) were included and were matched for age and BMI. All participants were Caucasians. We excluded participants with previously diagnosed diabetes and other endocrine disorder, cancer, and other major illness, recent history (past 12 months) of drug abuse or alcohol abuse, as well as ongoing medication with either of neuropsychiatric stimulants, beta-blockers, antipsychotics, systemic glucocorticoids, or other immune-modulating drugs and use of CPAP or oral appliances. None of the participants with OSA were active smokers. In the group without OSA, only one participant was an active smoker.

### Polysomnography

All the men underwent overnight ambulatory polysomnography (EMBLA, Flaga Inc., Iceland) including continuous 16-channel recordings of two airflow leads (oronasal three-port thermistor and nasal flow pressure sensor), two respiratory effort leads (thoracic and abdominal) from piezoelectric belts (Resp-EZ; EPM Systems, Midlothian, VA, USA), one pharyngeal sound lead (from a piezo vibration sensor), finger pulse oximetry (Embla A10 flex Sensor), two electroencephalography leads (C3-A2 and C4-A1), two electrooculography leads, three electromyography leads (sub-mental, left and right anterior tibialis muscles), two electrocardiography leads, and one body position lead.

Sleep was scored manually in 30-s periods [19, 20]. Obstructive apnea was defined as the complete cessation of nasal and oral airflow lasting 10 s or more with continuing abdominal and thoracic movements. Hypopnea was defined as a ≥50% reduction in both oronasal thermistor and nasal pressure for at least 10 s compared with baseline, followed by an oxygen desaturation of ≥3% or arousal. The AHI was defined as the mean number of apneas and hypopneas per hour of sleep. The REM-AHI was calculated as the number of apneas and hypopneas during REM sleep divided by the hours spent in REM sleep.

### Anthropometric and biochemical measurements

Participants were recruited as with OSA or without OSA based on AHI scores and were investigated under normal conditions in the morning after overnight fasting, i.e., regardless of OSA episodes the preceding night. All participants underwent a detailed investigation of their medical history and a physical examination, including height (cm), weight (kg), waist (cm), and hip (cm) circumference, which were carried out according to the World Health Organization guidelines. Body composition was determined by bioelectrical impedance (Kroppsanalysator BC-418MA, Tania). The lipid profile (triglyceride, total cholesterol, high-density cholesterol, and low-density lipoprotein were analyzed from a fasting blood sample. Homeostatic model assessment of insulin resistance (HOMA-IR) was calculated using fasting plasma glucose and insulin values [21]. All blood plasma and serum samples were analyzed at the Department of Clinical Chemistry, Uppsala University Hospital.

### Oral glucose tolerance test (OGTT)

A 2-h oral glucose tolerance test (OGTT) was performed after the consumption of a 75-g glucose solution. Blood samples were collected at baseline and 15, 30, 60, 90, and 120 min after glucose consumption. From the OGTT, glucose, insulin, total glucagon-like peptide-1 (GLP-1), nonesterified fatty acids (NEFA), and glycerol were analyzed. NEFA was measured using NEFA fluorometric assay kit (Cayman Chemicals, Ann Arbor, MI, USA). Glycerol quantification was done using free glycerol reagent (Sigma chemicals Co., St. Louise, MO, USA) and plasma GLP-1 was measured using a commercial kit (#EZHGIP54K; Merck Millipore, Darmstadt, Germany). The glucose, insulin, and NEFA levels obtained during OGTT were used to calculate insulin sensitivity indices (ISI), which were determined by the Matsuda index [22], Gutt index [23], revised quantitative insulin sensitivity check (QUICKI) [24], insulinogenic index (IGI) [25], and disposition index (DI) [26]. The adipose tissue insulin sensitivity index (ISI) (FFA) was estimated using the method described previously [27].

The following formulas were used to calculate different ISI:

$$\begin{array}{l}{\rm{Revised \ QUICKI}}\\ = \frac{1}{{\left( {{\log} \,{\rm{fasting}}\,{\rm{glucose}} \,+\, {\log} \,{\rm{fasting}}\,{\rm{insulin}} \,+\, {\log} \,{\rm{fasting}}\,{\rm{NEFA}}} \right)}}\end{array}$$

$$\begin{array}{l}{\rm{Matsuda}}\,{\rm{Index}} \\ = \frac{{10,000}}{{\sqrt {{\rm{fasting}}\,{\rm{glucose}}\,\times\,{\rm{fasting}}\,{\rm{insulin}}\,\times\,{\rm{mean}}\,{\rm{OGTT}}\,{\rm{glucose}}\,\times\,{\rm{mean}}\,{\rm{OGTT}}\,{\rm{insulin}}} }}\end{array}$$

$${\rm{Disposition}}\,{\rm{Index}} = \frac{{\delta {\rm{insulin}} \, \left( 0 - 30\,{\rm{min}} \right)}}{{\delta {\rm{glucose}} \, \left( 0 - 30\,{\rm{min}} \right)}}\times\frac{1}{{\rm{fasting}}\,{\rm{insulin}}}$$

$${\rm{IGI}} = \frac{\delta {\rm{insulin}} \ \left( 0 - 30\,{\rm{min}} \right)}{\delta {\rm{glucose}} \, \left( 0 - 30\,{\rm{min}} \right)}$$

$$\begin{array}{*{20}{l}}{{\rm{Gutt}}\,{\rm{Index}}}\\{ = \frac{{75,000 + \left( {{\rm{fasting}}\,{\rm{glucose}} - {\rm{glucos}}{{\rm{e}}_{120\,{\rm{min}}}}} \right) \times 0.19 \times {\rm{Body}}\,{\rm{weight}}}}{{120 \times \log \left[ {\frac{{{\rm{fasting}}\,{\rm{insulin}} + {\rm{insuli}}{{\rm{n}}_{120\,{\rm{min}}}}}}{2}} \right] \times \left[ {\frac{{{\rm{fasting}}\,{\rm{glucose}} + {\rm{glucos}}{{\rm{e}}_{120\,{\rm{min}}}}}}{2}} \right]}}}\end{array}$$

$${\rm{Stumvoll}}\,{\rm{ISI}} = 0.226 - 0.0032\,\times\,{\rm{BMI}} - 0.0000645\,\times\,I_{120} - 0.00375\,\times\,G_{90}$$

$${\rm{ISI}}\, \left( {\rm{ffa}} \right) = \frac{2}{\left( {\rm{INSp}}\,\times\,{\rm{NEFAp}} \right) + 1}$$

INSp and NEFAp were calculated from the AUC 0–60 min and 60–120 min for respective measurement.

### Heart rate variability (HRV) analyses

R–R intervals were determined from ECG:s recorded during 5 min in the supine position. All recordings were manually inspected to correct for detection errors and spurious extrasystolic beats. However, recordings with very frequent arrhythmic beats were excluded from the HRV analysis. The series of R–R intervals related to normal sinus rhythm was transformed to evenly sampled (2 Hz) data by cubic spline interpolation. HRV was analyzed by power spectrum analysis of R–R intervals using Welch’s periodogram method. The total spectral power (PTOT) and the power of three spectral components were calculated: the very low frequency (PVLF, fluctuations below 0.04 Hz), the low-frequency (PLF, 0.04–0.15 Hz), and the high-frequency (PHF, 0.15–0.50 Hz) component. The ratio PLF/PHF was used as a marker of the balance between sympathetic and parasympathetic activity since PHF mainly reflects the parasympathetic part, whereas PLF reflects a combination of sympathetic and parasympathetic activity. All spectral indices were log-transformed and calculated as averages over the complete 5-min recording. The HRV analysis was performed using Matlab software (MathWorks, Natick, MA).

### Statistical analysis

The sample size for this study was chosen to provide at least 80% power to detect a 20% difference in AUC glucose between groups, and this was based on the previous study of individuals with or without diabetes [28]. Analyses were performed assessing associations between an indicator variable representing a group (0 = control, 1 = case) and different outcome variables (including clinical characteristics and OGTT- and HRV-related measures) using linear regression. The analyses were made both crude and adjusted for BMI, age, and body fat.

It was also tested whether the difference from baseline was different for participants with OSA and participants without OSA for each time point during the OGTT test. Since the OGTT test measures each subject several times, we used linear mixed models with glucose, insulin, glycerol, FFA, and GLP-1 as outcome variables, using the subject as a random intercept. As explanatory variables, we used categorical variables for the group, time point, and interactions between group and time point.

All inference was made using bootstrapped standard errors. For linear mixed models, we used the cluster bootstrap. The bootstrap can be used for dealing with nonnormal data without having to resort to transformations or rank-based methods (like the Mann–Whitney test). Descriptive statistics are presented as mean ± standard deviation. The area under the curves (AUC) for OGTT tests was calculated using the trapezoid method. Statistical significance was defined as *p* < 0.05. Spearman’s correlation analysis was used to test the association between body fat% and clinical and metabolic variables. All analyses were performed using Stata 14.

## Results

### Anthropometric and clinical characteristics of participants

The anthropometric and clinical characteristics of the study participants are given in Tables 1 and 2. The two groups were well matched for their age and BMI (Table 1). The body fat percentage was higher in men with OSA than in men without OSA (*p* = 0.046), despite no difference in the body weight and waist circumference between the groups. Moreover, in men with OSA, the body fat% was positively associated with markers of insulin resistance such as fasting insulin (*ρ* *=* 0.745, *p* < 0.01), HOMA-IR (*ρ* *=* 0.708, *p* < 0.01), and negatively with markers of insulin sensitivity such as Matsuda index (*ρ* *=* −0.795, *p* < 0.001), Stumvoll ISI (*ρ* *=* −0.626, *p* < 0.05). Men with OSA had higher fasting plasma glucose than those without OSA (*p* < 0.001). The blood pressure also did not differ between groups (Table 1). In addition, C-peptide was higher in men with OSA than in control (*p* = 0.039; Table 2), while there was no significant difference in fasting serum insulin, HbA1c, and lipid profile between the groups (Table 2).

**Table 1:** Anthropometric characteristics of participants

Variable	Men without OSA (*n* = 15)	Men with OSA (*n* = 15)	*p* value
Age (years)	65 (8)	68 (8)	0.403
Weight (kg)	87.9 (12.5)	88.8 (13)	0.837
BMI (kg/m^2^)	27.6 (3.4)	27.8 (3.3)	0.867
Waist circumference (cm)	103.5 (10.5)	105.4 (9.1)	0.613
Waist-hip ratio	0.97 (0.06)	0.98 (0.05)	0.350
Body fat (%)	25.5 (4.9)	28.7 (3.6)	0.046
Systolic blood pressure (mmHg)	143 (19)	136 (19)	0.279
Diastolic blood pressure (mmHg)	90 (11)	85 (8)	0.191

Data are shown as means (SD). The *p* values are from crude linear regression analyses with bootstrapped standard errors

*BMI* body mass index

**Table 2:** Metabolic measures

Variable	Men without OSA (*n* = 15)	Men with OSA (*n* = 15)	*p* value
Fasting plasma glucose (mmol/l)	5.8 (0.3)	6.3 (0.4)	*p* < 0.001
Fasting serum insulin (mU/l)	8.0 (4.9)	8.6 (3.8)	0.548
Glucose, OGTT AUC_120 min_ (min × mmol/l)	938 (114)	1128 (119)	*p* < 0.001
Insulin, OGTT AUC_120 min_ (min × mU/l)	5241 (3340)	5404 (1893)	0.865
2-h plasma glucose (mmol/l)	7.0 (1.3)	8.8 (1.5)	0.001
Serum C-peptide (nmol/l)	0.7 (0.2)	0.9 (0.2)	0.039
HbA1c (mmol/mol, IFCC)	36.4 (2.4)	36.5 (2.7)	0.880
Fasting plasma triglycerides (mmol/l)	1.0 (0.4)	1.2 (0.4)	0.189
Fasting plasma HDL cholesterol (mmol/l)	1.3 (0.1)	1.3 (0.3)	0.901
Fasting plasma LDL cholesterol (mmol/l)	3.4 (0.9)	3.5 (0.7)	0.708
LDL/HDL	2.7 (0.8)	3.0 (1.0)	0.442
Fasting plasma NEFA (μM)	214 (77)	263 (87)	0.097
Fasting plasma glycerol (μM)	77 (29)	76 (23)	0.686
NEFA, OGTT AUC_120 min_ (min × μmol/l)	11,413 (3694)	14,905 (4633)	0.021
Glycerol, OGTT AUC_120 min_ (min × μmol/l)	6840 (2173)	7416 (2479)	0.475
Fasting plasma total GLP-1 (pmol/l)	17 (8.0)	18 (9.0)	0.864
GLP-1 total AUC_120 min_ (min × pmol/l)	2564 (1221)	2935 (1318)	0.413

Data are shown as mean (SD). Blood samples were obtained during fasting or OGTT as indicated. The *p* values are from crude linear regression analyses with bootstrapped standard errors

*HbA1c* glycated hemoglobin (HbA1c), measured in whole blood, *HDL* high-density lipoprotein, *LDL* Low-density lipoprotein, *NEFA* nonesterified fatty acids, *OGTT* oral glucose tolerance test

### Oral glucose tolerance test (OGTT)

**Fig. 1:**
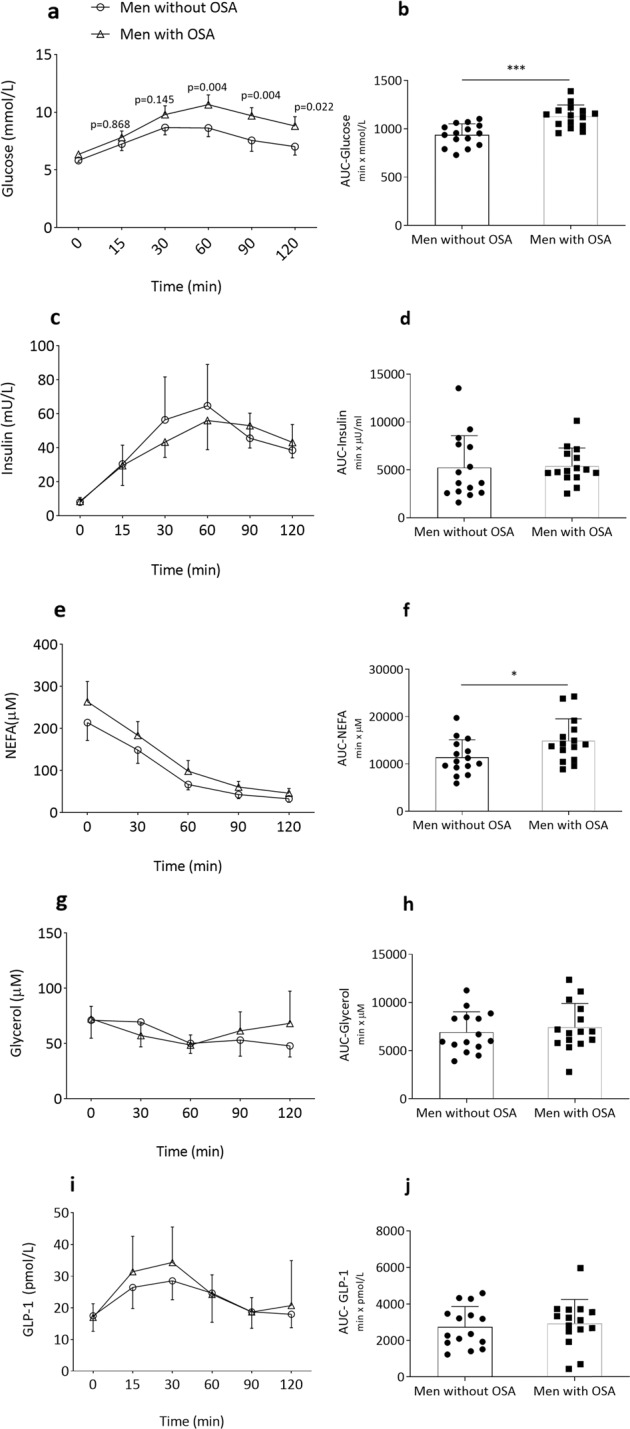
Average plasma concentrations and AUC for plasma glucose (**a**, **b**), serum insulin (**c**, **d**), NEFA (**e**, **f**), glycerol (**g**, **h**), and total GLP-1 (**i**, **j**) during a 2-h OGTT between men with OSA and men without OSA. NEFA nonesterified fatty acids, GLP-1 glucagon-like peptide-1. *p < 0.05; ***p < 0.001. Data are shown as mean ± SD

Men with severe OSA had significantly higher glucose concentrations during the OGTT than control participants (Fig. 1a, b). This was true for the 2-h glucose (*p* = 0.001) as well as the AUC for glucose (*p* < 0.001). Insulin levels or AUC for insulin during OGTT, on the other hand, did not differ between groups (Fig. 1c, d). NEFA levels at baseline were non-significantly higher in men with OSA vs. those without OSA (*p* = 0.097, Table 2). However, NEFA levels were less suppressed in men with OSA than without OSA during the OGTT (Fig. 1e). Thus, the total AUC for NEFA was higher in participants with OSA than without OSA (*p* = 0.021, Fig. 1f). The glycerol levels were similar at baseline and did not change during OGTT between groups (Fig. 1g, h). GLP-1 levels during fasting or following oral glucose did not differ between groups (Fig. 1i, j).

### Insulin sensitivity indices derived from fasting and OGTT sampling

**Fig. 2:**
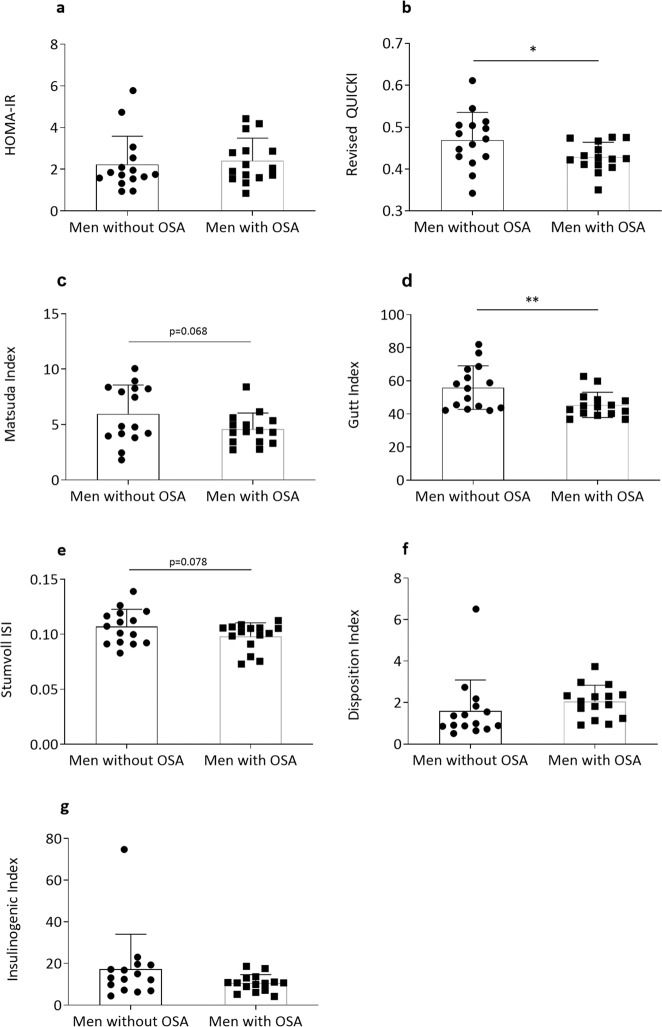
Fasting and OGTT-based indices for insulin sensitivity. HOMA-IR (**a**), Revised QUICKI (**b**), Matsuda index (**c**), Gutt index (**d**), Stumvoll ISI (**e**), Disposition index (**f**), and Insulinogenic index (**g**). HOMA-IR homeostatic model assessment for insulin resistance, ISI insulin sensitivity index, QUICKI quantitative insulin sensitivity check index. *p < 0.05; **p < 0.01. Data are shown as mean ± SD

Different indices of insulin sensitivity were determined using levels of glucose, insulin, and NEFA at either fasting or during OGTT. Results are summarized in Table 3 and Fig. 2. Revised QUICKI was significantly (*p* = 0.041) and HOMA-IR non-significantly (*p* = 0.697) higher in men with OSA than those without OSA. The OGTT-based Gutt index (*p* = 0.005) and Matsuda index (*p* = 0.068) were lower in men with OSA compared with without OSA. The IGI which is a marker of beta-cell function and DI were numerically but not significantly lower in participants with OSA vs. without OSA. The adipose tissue ISI for lipolysis was similar between groups (data not shown).

**Table 3:** Fasting and OGTT-based insulin sensitivity indices

Variable	Men without OSA (*n* = 15)	Men with OSA (*n* = 15)	*p* value
HOMA-IR	2.2 (1.4)	2.4 (1.1)	0.697
Revised QUICKI	0.5 (0.07)	0.4 (0.03)	0.041
Matsuda index	6.0 (2.6)	4.6 (1.5)	0.068
Gutt index	56.0 (13.2)	45.5 (7.6)	0.005
Stumvoll ISI	0.11 (0.01)	0.10 (0.01)	0.078
Insulinogenic index	17.2 (16.8)	10.5 (4.1)	0.744
Disposition index	2.0 (0.8)	1.6 (1.5)	0.285

Data are shown as mean (SD). The *p* values are from crude linear regression analyses with bootstrapped standard errors

*HOMA-IR* homeostatic model assessment for insulin resistance, *ISI* insulin sensitivity index, *QUICKI* quantitative insulin sensitivity check index

To assess whether REM-AHI contributes further to metabolic adverse effects, we compared anthropometric and metabolic variables between participants with REM-AHI < or >30 from the whole cohort (*n* = 19 and *n* = 11, respectively). However, we did not find any major difference for any variable between those groups (data not shown). Besides, among participants with OSA, when divided into two groups by the median REM-AHI, no major differences were observed for any of the metabolic variables assessed. However, when considered the whole cohort, in bivariate correlation analysis, the REM-AHI score was associated with fasting glucose (*ρ* = 0.606, *p* < 0.01) and AUC glucose during OGTT (*ρ* = 0.564, *p* < 0.01).

### HRV analysis results

The HRV data are shown in Table 4. Four recordings in Men with OSA and one recording in men without OSA were excluded due to very frequent extrasystoles. In addition, three recordings in the group without OSA were lost due to technical failure during the ECG recording. Thus, HRV could be analyzed in 11 men with OSA and 11 men without OSA. The total power and the power of the low-frequency component, respectively, were non-significantly higher in the men without OSA vs. with OSA (*p* < 0.10 for both), whereas the high-frequency component did not differ. There were no differences between groups in mean R–R and PLF/PHF.

**Table 4:** Heart rate variability measures

Variable	Men without OSA (*n* = 11)	Men with OSA (*n* = 11)	*p* value
R–R (S)	1.00 (0.09)	0.97 (0.09)	0.426
Ptot (ms^2^, log)	3.09 (0.36)	2.85 (0.32)	0.079
PLF (ms^2^, log)	2.61 (0.37)	2.32 (0.40)	0.070
PHF (ms^2^, log)	2.10 (0.46)	1.91 (0.43)	0.292
PLF/PHF (log)	0.41 (0.32)	0.50 (0.33)	0.475
Pvlf (ms^2^, log)	2.81 (0.39)	2.58 (0.30)	0.099

Data are means (SD). Spectral indices are log-transformed. The *p* values are from crude linear regression analyses with bootstrapped standard errors

*R–R* R–R interval, *Ptot* total power, *PLF* power of low-frequency component, *PHF* power of high-frequency component, *Pvlf* power of very low-frequency component

## Discussion

In this community-based study, we performed rigorous metabolic investigations of men with severe OSA compared with age- and BMI-matched men without OSA, defined as AHI >30 and <5, respectively. To assess direct associations between OSA and metabolic measures and to avoid confounding effects we included patients who had not yet started any active treatment for OSA, for example, the use of CPAP or oral appliances. Moreover, investigations were performed in the randomly selected mornings with no specified relation to OSA episodes.

We showed that despite matching for BMI, men with severe OSA had a higher body fat percentage than those without OSA. Thus, to test if the body fat percentage may be a factor contributing to insulin resistance in the OSA group, we tested its association with different biochemical variables and insulin sensitivity indices derived from them. We found that the body fat percentage was positively associated with markers of insulin resistance independent of BMI. Thus, this is not directly linked to obesity as the groups were matched for BMI, but it might be accompanied by fat redistribution between depots. Waist circumference and waist-hip ratio, the measures of abdominal adiposity and fat redistribution, were numerically but not significantly increased and there may be a visceral and ectopic fat accumulation. The relative fat accumulation, together with a potential redistribution, could contribute to the development of both OSA and its associated metabolic disturbances as observed in the present study. Alternatively, it could be a consequence of OSA, for example, mediated by neuroendocrine factors including the autonomic nervous system. Besides, men with severe OSA had elevated NEFA levels at fasting, as well as during OGTT compared with men without OSA.

Men with severe OSA showed impaired glucose tolerance, however, the incretin response was similar between the groups. ISI pointed toward insulin resistance in the OSA group having lower Matsuda, Gutt, Stumvoll ISI, and revised QUICKI compared with participants without OSA. The autonomic nerve activity determined using the heart rate variability measures was similar between groups.

### Fatty acid turnover

Men with OSA showed non-significantly higher NEFA at fasting compared with men without OSA but showed higher total AUC for NEFA during OGTT. However, the percent suppression of NEFA during OGTT did not differ and may suggest that adipose tissue insulin sensitivity was similar between groups. This could be further supported by the similar ISI for lipolysis (data not shown). Moreover, glycerol, which undergoes re-esterification into adipocytes to less extent than NEFA and thus better estimates lipolysis, also did not differ between groups. Previous research has shown increased NEFA levels also after adjustment for BMI and metabolic syndrome [29]. Surprisingly, one early CPAP treatment study in six patients with OSA showed significantly greater concentrations of NEFA on the treatment night. The authors proposed that the suppression of growth hormone and its lipolytic action in OSA is reversed by CPAP treatment [30]. Of note, in the present study, the higher NEFA levels in participants with OSA might be explained by its impaired utilization in the liver or muscle or re-esterification in adipose tissue [28].

### Incretin response

In the present study, we also assessed whether impaired GLP-1 levels at fasting or in response to OGTT could explain the impairment of glucose tolerance in OSA. GLP-1 is a peptide secreted in response to nutrients by L-cells located throughout the small intestine and colon. In patients with type 2 diabetes, GLP-1 levels are reduced in response to OGTT when compared with individuals without diabetes [31]. In the present study, however, we did not see any difference between men with and without OSA, suggesting that the differences in glucose tolerance between the two study groups might not be mediated by GLP-1. Few studies have previously assessed the associations between OSA and GLP-1; however, results from meal tolerance tests have shown that the severity of OSA was related to higher fasting GLP-1 [32], although this association was diminished after adjusting for the degree of adiposity. Another study demonstrated that increasing OSA severity was associated with lower GLP-1 response to glucose challenge [33]. Notably, these previous studies were performed in Asian males, limiting their generalizability to other ethnic groups. To the best of our knowledge, for the first time, we show that in Caucasian men OSA did not affect basal or stimulated GLP-1 levels. Nonetheless, it should be noted that we measured total GLP-1, and levels of active GLP-1 may potentially be different between groups. Besides, other incretin hormones such as GIP might be of importance.

### Glucose tolerance

The present study showed higher fasting plasma glucose and a reduced ability of insulin to counter glucose elevation during OGTT in men with severe OSA compared with those without OSA. This was seen throughout the OGTT despite similar insulin levels in both groups, suggesting an impaired action of insulin on endogenous glucose production by the liver (suppression of gluconeogenesis and glycogenolysis) and glucose disposal in peripheral organs such skeletal muscle and adipose tissue. This could be further supported by the observed differences in ISI such as Matsuda and Gutt, both being well-correlated with whole-body insulin sensitivity determined using a gold standard hyperinsulinemic–euglycaemic clamp. Previous population-based studies on OSA and glucose metabolism have shown OSA to be associated with decreased insulin sensitivity [11] derived from OGTT, insulin resistance, and impaired glucose tolerance [34], increasing HbA1c, and prediabetes [35]. Moreover, in 118 participants without diabetes, who underwent an intravenous glucose tolerance test, AHI was independently associated with insulin sensitivity, even after adjusting for age, sex, race, and body fat percentage [36]. Furthermore, that study also reported that the rapid insulin response to glucose did not increase with the severity of OSA, suggesting that OSA is related to insulin resistance with insufficient compensation by the beta cells [36]. The relationship between OSA and impaired glucose metabolism has also been shown in participants without obesity [37].

### Autonomic nerve activity

Elevated sympathetic nerve activity has been suggested to be associated with metabolic and cardiovascular risks in OSA [38]. To assess whether the autonomic nervous system tone is affected in men with severe OSA, we assessed the HRV between men with and without OSA. However, HRV components representing the change in either sympathetic or parasympathetic nerve activity did not significantly differ between groups. Nevertheless, the observed trends in the spectral components suggest a tendency to an overall reduction in both parasympathetic and sympathetic activity, resulting in an unchanged “autonomic balance” as supported by the unchanged LF/HF ratio and heart rate. Thus, our results do not point to the autonomic nervous system as the main factor for metabolic dysregulation in OSA. Importantly, we assessed HRV in the morning and not during sleep and OSA episodes, and thus autonomic responses during apnea episodes could contribute to long-term metabolic effects.

Patients with OSA are characterized by a high prevalence of arterial hypertension and high variability in their blood pressure [39]. In our study, we did not find any differences in either systolic or diastolic blood pressure between groups. This could be explained by frequent use of antihypertensive medications by patients with OSA (8 of 15 subjects vs 2 of 15 in the non-OSA group). Of note, blood pressure was measured in the morning in a resting but awake state and not when the subjects were asleep, possibly having OSA attacks, which may also have a bigger impact on blood pressure. In addition, obesity contributes to hypertension in OSA patients, and our data in BMI-matched subjects may suggest a small hypertensive effect of OSA itself.

### Limitations

The present study was performed in well-matched individuals that had been objectively assessed for OSA and were rigorously assessed for metabolic measures as well as autonomic nerve activity. However, there are limitations to consider when interpreting the results. Although well-matched, the study groups are small which increases the risk of being underpowered. Besides, only men were included thereby reducing the generalizability of the results. Moreover, all assessments, except the initial OSA monitoring, were performed in the morning under fasting and resting conditions with the participants being awake. During OSA attacks, acute responses may very well involve autonomic nerve, hormonal and metabolic pathways, and this was not addressed in this study. Nonetheless, it provides further understanding of the relationship between OSA and chronic metabolic dysregulation by thoroughly investigating insulin secretion, insulin sensitivity, glucose tolerance, fatty acid metabolism, as well as autonomic nervous activity. Furthermore, patients receiving CPAP treatment were not included, therefore results are only applicable in OSA without treatment. Lastly, we did not find a specific impact of REM sleep apnea on metabolic outcomes in our study. A recent study has shown that REM-AHI is associated with metabolic syndrome and diabetes [13]. Admittedly, our study probably has a too small sample size to explore the unique role of REM-OSA. Larger studies are needed to clarify the relation between REM-AHI and metabolic disease.

## Conclusion

In this cross-sectional study, we show that middle-aged, overweight men with OSA, compared with BMI- and age-matched men without OSA, have impaired glucose and fatty acid metabolism and also increased body fat percentage. These alterations can largely be explained by insulin resistance. Therefore, it is important to assess glucose and lipid metabolism in patients with OSA to be able to mitigate the risk for type 2 diabetes and cardiovascular disease.
